# Reference-based chromosome-scale assembly of Japanese barley (*Hordeum vulgare* ssp. *vulgare*) cultivar Hayakiso 2

**DOI:** 10.1093/dnares/dsaf016

**Published:** 2025-06-19

**Authors:** Tsuyoshi Tanaka, Yuhi Haraguchi, Takatomo Todoroki, Daisuke Saisho, Tomomi Abiko, Hiroomi Kai

**Affiliations:** Bioinformatics Unit, Research Center for Advanced Analysis, National Agriculture and Food Research Organization, Ibaraki 305-8518, Japan; Department of Crop Production and Breeding, Fukuoka Agriculture and Forestry Research Center, Fukuoka 818-8549, Japan; Department of Crop Production and Breeding, Fukuoka Agriculture and Forestry Research Center, Fukuoka 818-8549, Japan; Barley Germplasm Center, Institute of Plant Science and Resources, Okayama University, Okayama 710-0046, Japan; Laboratory of Agroecology, Graduate School of Bioresource and Bioenvironmental Sciences, Kyushu University, Fukuoka 819-0395, Japan; Department of Crop Production and Breeding, Fukuoka Agriculture and Forestry Research Center, Fukuoka 818-8549, Japan

**Keywords:** *Hordeum vulgare*, genome sequencing, long-read sequencing

## Abstract

Current advances in next-generation sequencing (NGS) technology and assembling programs permit construct chromosome-level genome assemblies in various plants. In contrast to resequencing, the genome sequences provide comprehensive annotation data useful for plant genetics and breeding. Herein, we constructed a reference-based genome assembly of winter barley (*H. vulgare* ssp. *vulgare*) cv. ‘Hayakiso 2’ using long and short read NGS data and barley reference genome sequences from ‘Morex’. We constructed ‘Hayakiso 2’ genome sequences covering 4.3 Gbp with 55,477 genes. Comparative genomics revealed that 14,106 genes had orthologs to two barley data, wheat (A, B, and D homoeologs, respectively), and rice. From the gene ontology analysis, 2,494 orthologs against wheat and rice but not two barley contained agricultural important genes, such as ‘response to biotic and abiotic stress’ and ‘metabolic process’. Phylogenetic analysis using 76 pangenome data indicated that ‘Hayakiso 2’ was clustered into Japanese-type genomes with unique alleles. ‘Hayakiso 2’ genome sequences showed known genes related to flowering and facilitated barley breeding through the development of various markers related to agronomically important alleles such as tolerance to various types of biotic and abiotic stress. Therefore, ‘Hayakiso 2’ genome sequences will be used for the further barley breeding.

## Introduction

Twenty years after the release of the rice genome sequences in 2004,^1^ > 5,000 of plant genome sequences are publicly available (https://www.ncbi.nlm.nih.gov/datasets/taxonomy/33090/). Although many genome assemblies were draft sequences, hundreds of chromosome-level genome assemblies have been released in Plant Ensembl (http://plants.ensembl.org/index.html) and Plant GARDEN (https://plantgarden.jp/ja/index).^2^ The construction of these accurate genome sequences was accelerated by developing long-read next-generation sequencing (NGS), super scaffolding methods such as high-throughput chromosome conformation capture (Hi–C), optical mapping, and assembly programs. By combining these technologies, we can target large and complicated plant genomes,^3–8^ such as 4.9 Gbp of barley genomes with seven chromosomes and 16.0 Gbp of wheat genomes with 21 chromosomes.^9^ Therefore, the size and complexity of genomes are not issued for genomic research and the application of genome analysis toward breeding and genome diversity becomes the current topic.^10,11^

Genome assembling strategies were *de novo* assembly and reference-based assembly. While *de novo* assembly was performed using the combination of long NGS reads, short NGS reads and genome structure analysis such as Hi-C and optical mapping, the reference-based assembly was conducted using reference genome sequences. While the reference-based assembly cannot detect the differences in genome structures like inversions and translocations, it provides comprehensive genomic data, for example, various annotation data including gene structures and physical positions, different from resequencing. Therefore, there are reports of reference-based assembly in plants.^12,13^

‘Hayakiso 2’, a winter barley (*H. vulgare* ssp. *vulgare*), was reported as a cultivar having an allele for early maturity used for the detection of barley *phytochrome C* (*HvPhyC*), a key factor in controlling long-day flowering.^14^ In addition to various phenotypic traits (http://earth.nig.ac.jp/~dclust/cgi-bin/barley_germ/detail_data.cgi?lang=ja&ID=132),^15^ ‘Hayakiso 2’ is tolerant to waterlogging^16^ and diseases such as barley yellow mosaic virus (BaYMV),^17^ being a highly relevant bioresource. Therefore, we considered that the genomic information of ‘Hayakiso 2’ can help accelerate not only the efficient development of new varieties but also clarify the biological mechanisms underlying biotic and abiotic stress tolerance.

To understand comprehensive genomic information and apply the genome data to the further breeding program, we constructed a reference-based assembly of ‘Hayakiso 2’ using a barley reference genome assembly from ‘Morex’. While this assembling procedure cannot detect the difference of genome structures against the reference genome, a comprehensive genome annotation data, such as genes, RNA genes, repeats, telomeres and centromeres, is available. By conducting comparative genome analysis using closely related species, wheat and rice, or barley pan genome data, we analysed the phylogenetic relationship and the genomic diversity.

## Materials and methods

### Sample preparation and sequencing

High-molecular-weight DNA (≥ 40 kb) was isolated from young leaves of ‘Hayakiso 2’ seedlings using the cetyltrimethylammonium bromide (CTAB) method.^18^ For long-read sequencing, genomic DNA was subjected to long-read library preparation using BluePippin (Sage Science, Beverly, Massachusetts, United States) with SMRTbell Express Template Prep Kit 2.0 (Pacific Bioscience, Menlo Park, California, United States). Fractionated DNA libraries 15–20 Kb in length were sequenced on a Sequel II system (Pacific Bioscience, Menlo Park, California, United States) by Kazusa DNA Research Institute. For short-read sequencing, library preparation using TruSeq DNA PCR Free from the DNA sample and sequencing by NovaSeq 6000 system with 150 bp of paired ends were outsourced to Macrogen Japan Corp. The newly generated raw sequence reads in this study are available at the DDBJ DRA database under run accessions (DRR619721–DRR619722).

### Construction of genome assembly and quality evaluation

High Fidelity (HiFi) reads were constructed by Circular Consensus Sequencing (commit v6.0.0-2-gf165cc26) and assembled by hifiasm (0.16.0).^19^ We downloaded ‘Morex’ genome assemblies (Version. 3 of release 52) from Ensembl Plants (https://plants.ensembl.org/Hordeum_vulgare/Info/Index).^20^ Reference-based scaffolding using ‘Morex’ genomes was performed using RagTag (v2.1.0).^21^ Polishing of the assembled sequences was performed using minimap2 (Release 2.10-r761)^22^ and hypo (v1.0.3) based on Illumina reads.^23^ The quality of the genome assembly was evaluated by Benchmarking Universal Single-Copy Orthologs (BUSCO) (version 5.7.1) with poales_odb10 (v. 2.1.0).^24^ Chromosome-level alignments between ‘Hayakiso 2’ and ‘Morex’ were constructed using the online tool D-Genies (https://dgenies.toulouse.inra.fr/).^25^

### Repeat masking and genome annotation

We downloaded the complete transposable elements platform (TREP) nucleotide sequences (v. 2019) comprising 4,162 sequences (https://trep-db.uzh.ch/)^26^ for repeatmasking ‘Hayakiso 2’ genome assembly using repeatmasker (4.1.2-p1).^27^ We surveyed a telomeric sequence motif, ‘TTTAGGG’ or similar motifs 1 bp different from the telomeric sequence using our own Perl scripts. The frequency was count in 10M bp window size. We searched for homologous sequences of HVT01 (GenBank Accession: X16095.1) and pAS1 (GenBank Accession: D30736.1) by BLASTN^28^ with the following threshold: Evalue < 1E^-5^. To detect centromere, we searched homologous regions of the Ty3/gypsy-like retrotransposon cereba sequence (GenBank Accession: AY040832.1) by BLASTN. We calculated the frequency of the homologous regions of pAS1 and cereba in 10M bp window size, respectively. For gene prediction, we used braker3 with Morex proteomes^29^ and Helixer (v0.3.3) with default parameters.^30^ The quality of the annotation data was evaluated by BUSCO (version 5.7.1) with poales_odb10 (v. 2.1.0).^24^ To assign functional annotations, we used InterProScan (interproscan-5.68-100.0)^31^ and extracted the InterPro domains and gene ontology (GO) terms from the results. We summarized the GOs in three functional categorizations (Biological Process, Cellular Component, and Molecular Function) by GOslim on Agbase (https://agbase.arizona.edu/).^32^ For tRNA annotation, tRNAscan-SE (2.0.12) was performed using default parameters.^33^ We ignored tRNA pseudogenes. For detecting rRNA regions, we performed a homology search of rRNA genes (GenBank Accession: KM217265.1) by BLASTN.

### Homology search and gene mapping

The homology search of ‘Hayakiso 2’ genes against four annotation data, ‘Morex’ Version 3 of release 52^20^ and ‘Haruna Nijo’ (pseudomolecules v1)^8^ for barley, ‘Nipponbare’ IRGSP-1.0^34^ for rice, and ‘Chinese Spring’ IWGSC refseq 2.1^35^ for wheat, was performed using BLASTP with the following threshold: Evalue < 1E^-5^. Orthologous gene sets were constructed using the reciprocal best hits. Codon-based alignments for barley orthologs among ‘Hayakiso 2’, ‘Morex’ and ‘Haruna Nijo’ were constructed using mafft-linsi (v7.525).^36^ Then, the nucleotide distance was calculated by dnadist of phylip-3.696 with Kimura’s two-parameters model.^37^ ‘Hayakiso 2’ genes were mapped on ‘Morex’ genome sequences with > 50% identity and coverage using GMAP.^38^

For constructing a phylogenetic tree, we conducted a homology search of ‘Hayakiso 2’ genes against 76 barley pangenome annotation data of barley cultivars.^3^ Using BLASTN, we determined the best homologues for ‘Hayakiso 2’ genes. Next, we aligned each protein sequence using mafft-linsi (v7.525) and reliable alignments with > 80% of identity between the ‘Hayakiso 2’ gene and each homolog were selected. After concatenating the alignments, a phylogenetic tree was constructed using MEGA12^39^ with the Neighbor-Joining^40^ and the Poisson correction methods,^41^ as well as a model of a gamma distribution (shape parameter = 2.25) as rate variation among sites.

## Results and discussion

### Construction of ‘Hayakiso 2’ genome assembly v.1.0

**Fig. 1.**
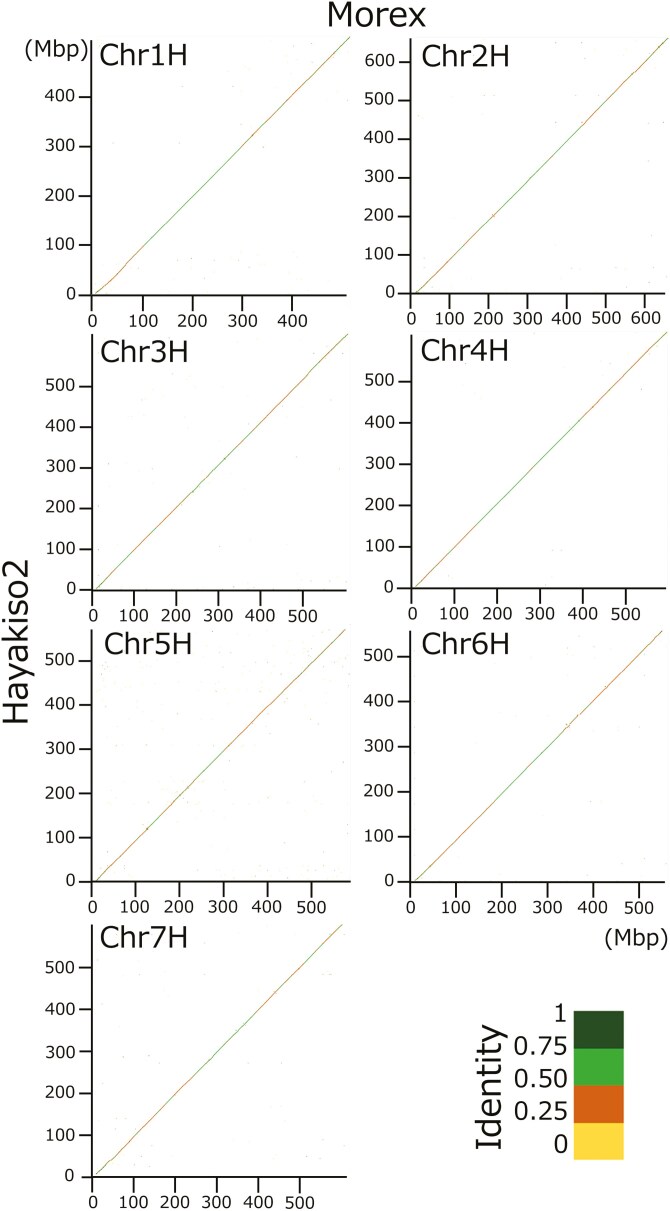
Chromosome alignments between ‘Hayakiso 2’ and ‘Morex’. X and Y axis represent ‘Morex’ and ‘Hayakiso 2’ genome sequences, respectively.

We conducted *de novo* assembling by hifiasm using 3,914,896 HiFi reads, reference-based scaffolding by RagTag, and polishing scaffolds by hypo using short-read NGSs (Supplementary Table 1). As a result, we constructed 3,584 scaffolds (Table 1). The seven longest sequences, comprising 4.2 Gb in length, corresponded to each barley chromosome (1H–7H) without large deletions under the chromosome alignment (Fig. 1). Given the scaffolding strategy, we could not evaluate known differences in genome structures, such as inversion on 2H, 4H, and 5H between ‘Morex’ and ‘Haruna Nijo’.^8^ BUSCO analysis showed improved assembly quality by polishing with Illumina reads (97.7%–98.5% in ‘Complete BUSCO’; Table 2). In addition, our assembly is comparable to the current version of ‘Morex’ genome sequences under BUSCO.

**Table 1.** Characteristics of assembled sequences.

	*De novo* assembly^*^	Reference-based scaffolding^*^	Polishing^*^	Morex (v3) Reference
Number of scaffolds	11,628	3,584	3,584	291
Total bases (bp)	4,338,869,646	4,339,674,046	4,339,452,547	4,225,577,519
Total bases (bp) (7 chromosomes)		4,200,652,824	4,200,446,802	4,196,495,466
Number of gap regions^**^			8,037	
Number of Ns (bp)^**^			803,700	

^*^‘de novo assembly’, ‘Reference-based scaffolding’, and ‘Polishing’ reprint the results from hifiasm, RagTag, and hypo, respectively.

^**^Gapped regions were inserted automatically by RagTag with 100 ‘Ns’.

**Table 2.** Genome completeness by BUSCO in’Hayakiso 2‘and’Morex’.

Genome	HKS2 (after polishing)	%	Morex (rel152)	%
Complete BUSCOs (C)	1,587	98.3	1,587	98.3
Complete and single-copy BUSCOs (S)	1,526	94.5	1,538	95.3
Complete and duplicated BUSCOs (D)	61	3.8	49	3.0
Fragmented BUSCOs (F)	16	1.0	18	1.1
Missing BUSCOs (M)	11	0.7	9	0.6
Gene				
Complete BUSCOs (C)	1,569	97.3	1,515	93.9
Complete and single-copy BUSCOs (S)	1,497	92.8	1,354	83.9
Complete and duplicated BUSCOs (D)	72	4.5	161	10.0
Fragmented BUSCOs (F)	28	1.7	13	0.8
Missing BUSCOs (M)	17	1.0	86	5.3

### Evaluation of the genome assembly

**Fig. 2.**
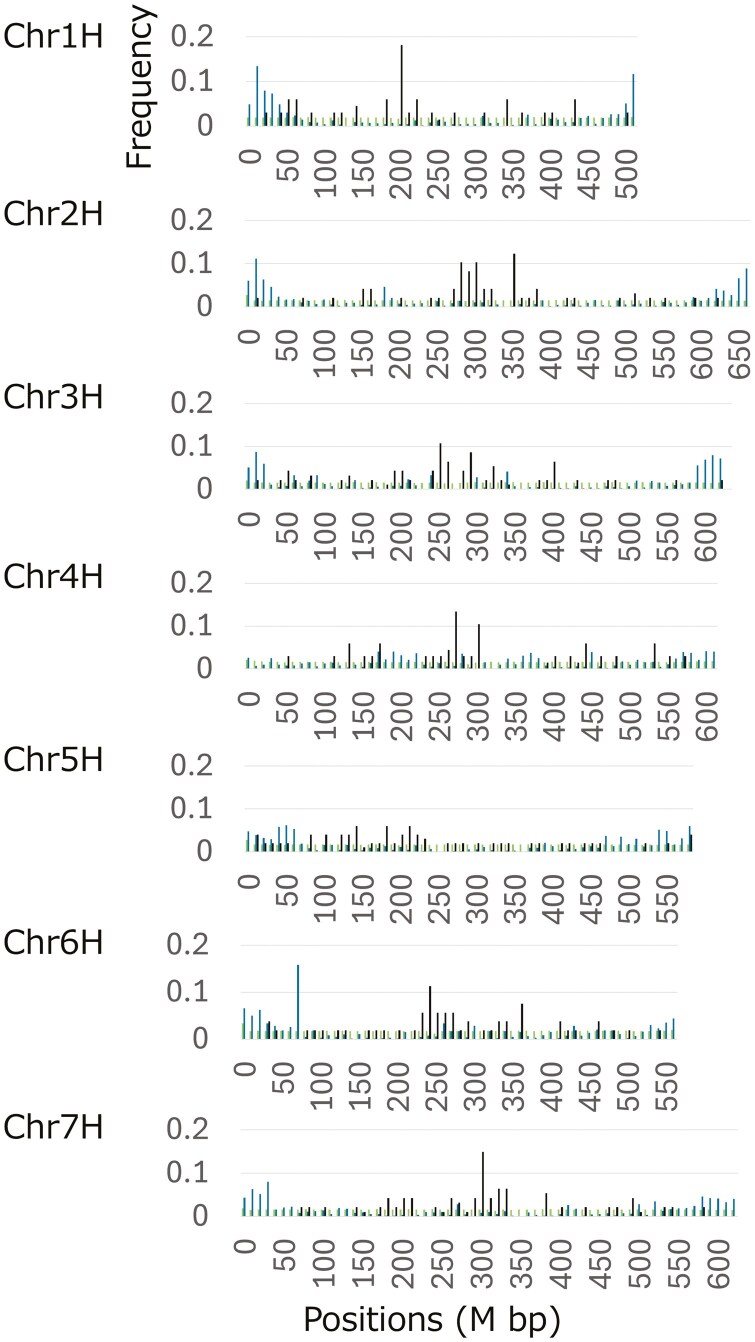
Distribution of TTTAGGG and its one-base mismatched repeat units (light blue), of pAS1 homologous units (light greem) and of cereba homologous units (black). The frequency of the homologous regions was calculated in 10M bp window size.

Based on previous studies,^42–44^ we defined the telomeric regions on our assembly using two methods. First, we searched for a telomere motif ‘TTTAGGG’ in ‘Hayakiso 2’ v.1 and detected dense regions of ‘TTTAGGG’ and the 1-bp mismatch sequences of ‘TTTAGGG’ on 5′-terminal regions of Chr2H, Chr5H, and Chr6H (Fig. 2). Second, we conducted a homology search of two subtelomeric sequences, HVT01 and pAS1. Both sequences were reported as associated sequences to barley telomeres but their characteristics were different.^43^ For HVT01, we detected only 245 homologous regions. While 67 hits were on scaffolds (nonchromosomal information), 178 concentrated on both ends of all chromosome sequences (104,074,794 bp from 3′-regions on Chr3H was the most distant from the end). In contrast, we found many hits for pAS1 (82,539 hits). Except for both ends of Chr4H and the 3′-end of Chr6H, 19 of 21 of the top 5% of the most hit regions (*N* = 423 regions) were located on the end or close regions (within 50 Mbp) of chromosomes (Fig. 2). Altogether, these results suggested that our assembly captured subtelomeric regions on almost chromosomes.

Next, we searched the centromeric regions of each chromosome. We mapped the Ty3/gypsy-like retrotransposon cereba sequence to the genome assembly,^45^ and observed that all chromosomes except for Chr5H had peaks of homologous hits, Chr1H: 210M bp, Chr2H: 360M bp, Chr3H: 260M bp, Chr4H: 280M bp, Chr6H: 250M bp, and Chr7H: 310M bp (Fig. 2). On Chr5H, there were three small peaks (150, 190, and 220 M bp). Since the homologous regions of the retrotransposon sequence were distributed broadly, we could not define the centromeric regions accurately. Combined with the linkage map from breeding studies, the information would be improved.

### Annotation of ‘Hayakiso 2’ genome assembly v.1.0

We detected repeat content using TREP information. A total of 82.8% of genome sequences were defined as known repeats, with Gypsy, Copia, and CACTA transposons being the top repeats (Supplementary Fig. 1). On the genome sequences, braker3 and Helixer predicted 126,624 and 55,477 genes, and the BUSCO analysis showed much higher values (97.3%) for Helixer than (27.9%) for braker3. We found that Helixer predicted many short exons (1–10 bp) (10.6% of total predicted exons), and braker3 predicted several shorter genes (> 200 amino acids in length), comprising 66.4% of total predicted genes. Based on the results, we adopted the annotation data of Helixer in this study and proceed to evaluate it. In total, 11,650 genes with short exons were not orthologs for four counterparts (two barley cultivars, ‘Morex’ and ‘Haruna Nijo’, rice and wheat homoeologs from A, B, and D genomes) and we consider that the orphan genes might cause of the different predicted gene number between ‘Hayakiso 2’ (55,477) and ‘Morex’ (37,961). In general, the genes have been distinguished into two groups by reliability, annotated and predicted in rice,^34^ and high and low confidences in wheat.^35^ However, the definitions of the reliability of the annotated genes were diversified, such as expression evidence, gene conservation, the existence of functional domains, and intron-exon structures. To avoid confusions, we did not distinguish annotated genes as high and low confidence genes and remained annotated genes by Helixer in our data.

Alternatively, to evaluate the predicted genes, we conducted comparative genomics analyses. First, we compared the distribution of gene functions between ‘Hayakiso 2’ and ‘Morex’. For functional annotation, we assigned the InterPro domains and GO information to the predicted genes. The distribution of GO terms was comparable to that of ‘Morex’ (Supplementary Fig. 2). GOslim analysis showed 2,027 genes with either GO:0006950 (response to stress), GO:0009607 (response to biotic stimulus), or GO:0009628 (response to abiotic stimulus). These genes might be related to the various stress tolerances of ‘Hayakiso 2’.

Second, we compared genes with other annotation data from barley, rice, and wheat. For barley, 44,748 (80.7%) and 47,004 (84.7%) of ‘Hayakiso 2’ genes were respectively homologous to ‘Morex’ and ‘Haruna Nijo’ genes; thus, 25,186 and 26,588 ortholog pairs were constructed based on the reciprocal best hits. A total of 98.1% of orthologs were located on the same chromosomes in ‘Hayakiso 2’ and ‘Morex’. Of 23,909 orthologous genes among the three species, 6,762 genes were completely identical at the nucleotide level. We narrowed the analysis of annotation data down to particular genes. When we focussed on known genes in ‘Hayakiso 2’, *HvPhyC* (AB827939) and *FT* (EF055987) were identical to HORVU.HK2.r1.5HG0027080.1 and HORVU.HK2.r1.7HG0069360.1, respectively. We confirmed a known amino acid change in *HvPhyC* of HORVU.HK2.r1.5HG0027080.1 from the ortholog analysis using ‘Morex’ and ‘Haruna Nijo’ (pSer440Phe).^14^ Moreover, *FT* was identical in ‘Hayakiso 2’, ‘Morex’, and ‘Haruna Nijo’. We found 66,849 ‘Hayakiso 2’ specific amino acid changes on 10,770 orthologs; these data would be applicable for future breeding programs. We also compared ‘Hayakiso 2’ genes with those of rice (RAP-DB annotation for IRGSP-1.0) and wheat (IWGSC refseq 2.1). In total, 49,649 (89.5%) genes had homologous sequences to ≥ 1 annotation data (two, one, and one in barley, wheat, and rice, respectively) and 14,106 genes had orthologs to two barley data, wheat (A, B, and D homoeologs, respectively), and rice. These data could help predict the evidence-based functional annotation of genes for forward genetics (Supplementary Table 2). For example, 2,494 genes were had orthologs against wheat and rice but not two barleys. From the GO slim analysis, we found that these genes contained dozens of stress response genes. In the homology search, 5,828 ‘Hayakiso 2’ genes were orphans. To evaluate ‘Hayakiso 2’ specific genes, we directly mapped 55,477 genes to ‘Morex’ genome sequences. For 53,611 genes, 96.6% were mapped to any genomic regions. Even if the threshold changed to 90% coverage or identity, 52,075 (93.9%) and 53,410 (96.3%) genes remained. We added all annotated data (InterPro domains, GOs, Orthologs, and nucleotide distance) to the GFF file.

We expanded the homology search to barley pangenome data.^3^ First, 48,755 of 55,477 (87.9%) ‘Hayakiso 2’ genes were homologous to some genes in 76 pangenome annotation data. Of 6,722 nonhomologous genes, 3,403 and 2,706 were short (< 100 amino acids in length) and short exon-containing (< 10 bp in length) genes, respectively. Therefore, these genes might not be specific genes but the orphan genes. Second, to analyse the evolutionary diversity of barley, we constructed a phylogenetic tree using virtual ortholog set based on 77 barley genomes. From the homology search and alignment analysis, we constructed 6,708 1-to-1 gene sets between ‘Hayakiso 2’ and other 76 barley pangenome data. Then, we concatenated the protein alignments and constructed a combined gene tree (Supplementary Fig. 3). Previously, the wild barley group initially diverged from the cultivated/landrace barley group.^3^ Our result also showed divergence; ‘Hayakiso 2’ clustered into the Japanese cultivar group including ‘CHIKURIN IBARAKI’ and ‘AKASHINRIKI’. Different clusters of ‘GOLDEN MELON’ and ‘AIZU 6’ might correspond to those previously reported.^3^ Therefore, we confirmed that ‘Hayakiso 2’ is a unique cultivar in Japan with diversified alleles.

To expand the genome annotation, we predicted RNA genes and found that our genome assembly did not contain tRNAs for particular codons (TGT for Cys, GGT for Gly, AGT for Ser, GAT for Asp, and CGC for Arg). The existence of lacked tRNAs on the genome sequences were observed in the barley pangenomes and rice genomes (10/61 tRNAs based on the IRGSP-1.0); thus, we considered that these codons were compensated by alternative tRNAs. We also predicted rDNA regions using a homology search. In barley genome, these were located on Chr3H and Chr5H.^11^ A homology search showed hits on six chromosomes except for Chr3H and 816 unassembled contigs. In total, 50 of 53 hits on Chr5H were located between 52.8M and 53.4M bp and 51 of 53 hits on Chr1H were located between 130.3M and 131.0M bp. The locations of these rDNA regions resulted from reference-based assembly using Morex genomes in which three rDNA regions were located (132.1M–132,8M bp on 1H, 52.6M–53.8M bp on 5H, and 81.9M–82.4M bp on 6H). With the possibility, because the many rDNAs were found on unassembled contigs, our current genome assembly lacked accurate rDNA regions.

## Conclusion

We constructed a reference-based genome assembly of ‘Hayakiso 2’ with a quality comparable to known barley reference genomes from ‘Morex’ and ‘Haruna Nijo’. Various genomic annotation including proteins, RNA genes, telomeric regions such as subtelomeric sequences, centromeres, and repetitive regions, can accelerate research into the phenotypic characteristics of ‘Hayakiso 2’. Annotating data with the physical genomic positions also helps in future accurate breeding in barley, such as marker assisted selection by constructing genome wide markers. In contrast, our annotation data contained short exon (< 10 bp in length) containing genes. Similar to the change in Morex gene number from 37,961 in MorexV3^17^ to 54,107 in the data published in 2024,^3^ future experimental research can update the annotation data of ‘Hayakiso 2’. Genome sequences and annotation data are available from DDBJ (accession numbers: BAAGGS010000001-BAAGGS010003584) and the Plant GARDEN (https://plantgarden.jp/en/list/t4513).^2^

## Supplementary Material

dsaf016_suppl_Supplementary_Figures_S1-S3

dsaf016_suppl_Supplementary_Table_S1

dsaf016_suppl_Supplementary_Table_S2
