# Supplementary material for: Reference-based chromosome-scale assembly of Japanese barley (Hordeum vulgare ssp. vulgare) cultivar Hayakiso 2
Source: DNA Res. 2025 Jun 19;32(4):dsaf016. doi: 10.1093/dnares/dsaf016 (PMC12232906; doi:10.1093/dnares/dsaf016)
Supplement: dsaf016_suppl_Supplementary_Figures_S1-S3 [file dsaf016_suppl_supplementary_figures_s1-s3.pdf]

Supplementary figures

Supplementary figure 1

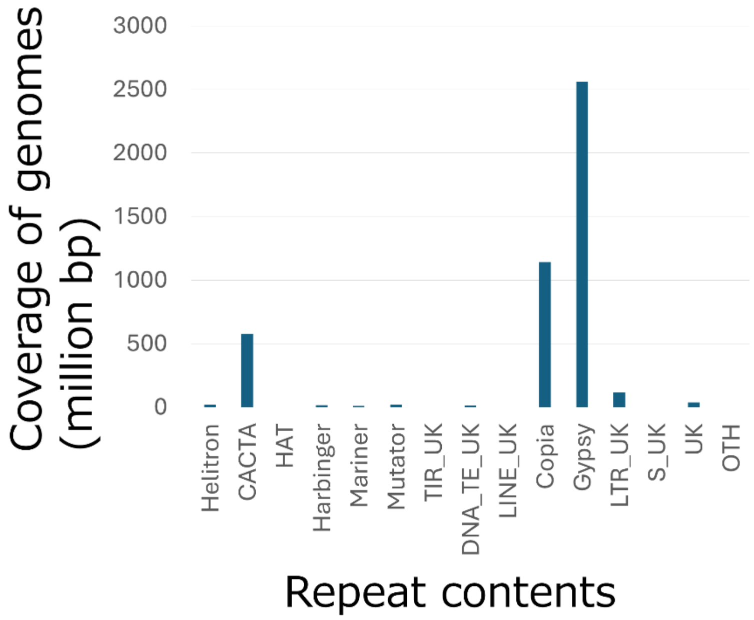

Supplementary Figure 1 Repeat contents of 'Hayakiso 2' genomes. Repeat names were derived from TREP library. Coverage of genomes was sum up the repetitive regions on the genome sequences detected by repeatmasker.

Supplementary figure 2

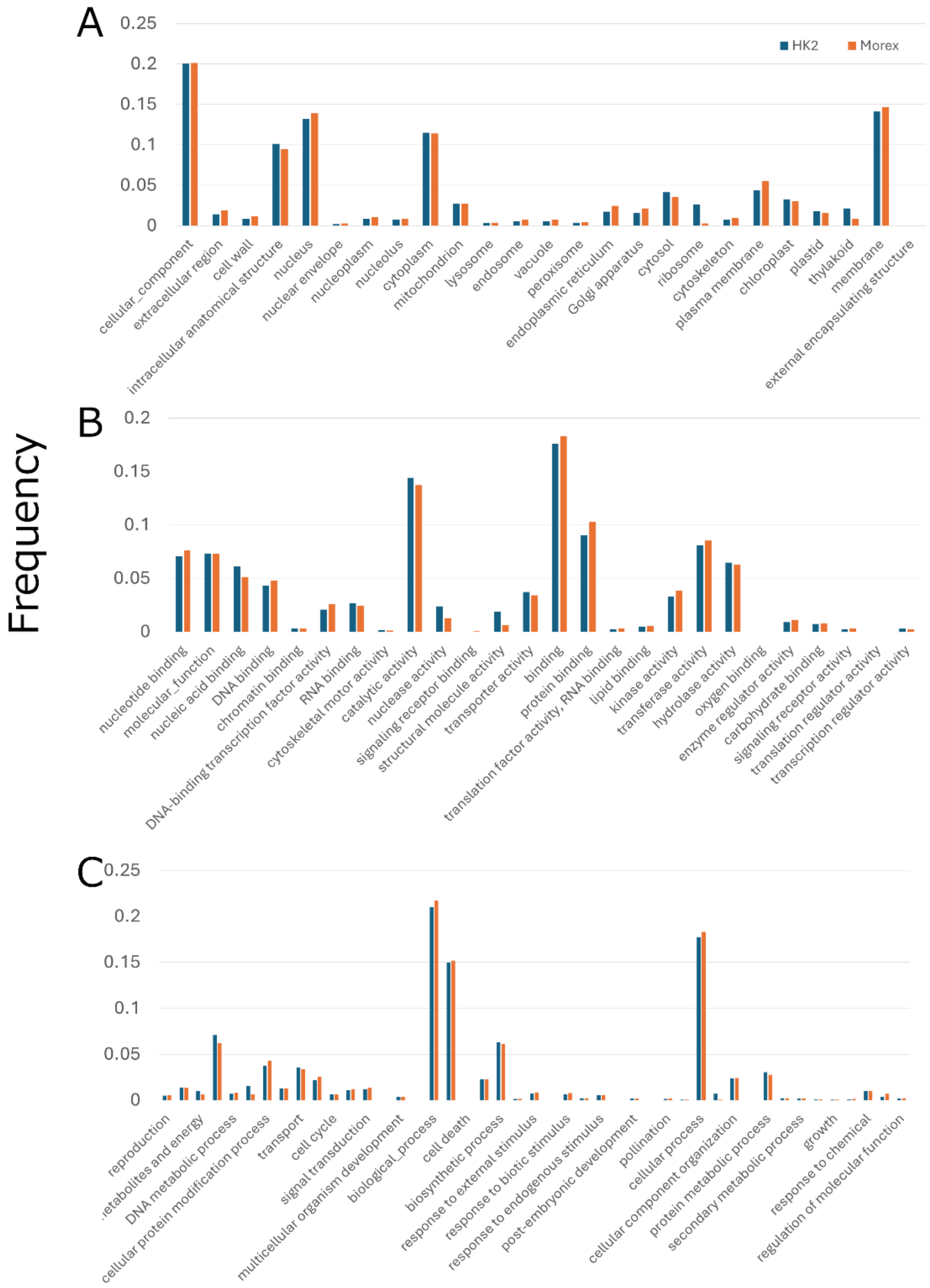

Supplementary Figure 2 Distribution of GO terms of, A) Cellular Component, B) Molecular Function and C) Biological Process, based on GOslim. GO terms were derived from the results of GOslim. Relative frequency was calculated in each GO category (Cellular Component, Molecular Function and Biological Process).

Supplementary Fig. 3

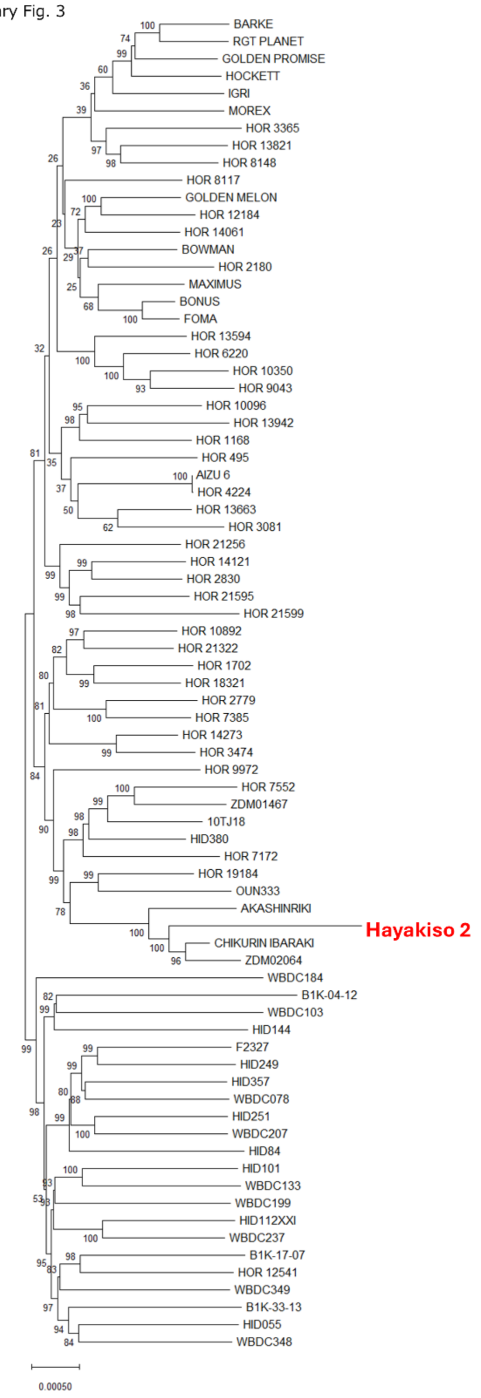

Supplementary Figure 3 Phylogenetic tree of 77 barleys. 6,708 ortholog groups were aligned independently and the alignments were concatenated into one alignment. The percentage of replicate trees in which the associated taxa clustered together in the bootstrap test (1,000 replicates) are shown next to the branches. The complete deletion option was applied to eliminate positions containing gaps and missing data resulting in a final data set comprising 3,056,386 positions.
