# Supplementary material for: Reference-based chromosome-scale assembly of Japanese barley (Hordeum vulgare ssp. vulgare) cultivar Hayakiso 2
Source: DNA Res. 2025 Jun 19;32(4):dsaf016. doi: 10.1093/dnares/dsaf016 (PMC12232906; doi:10.1093/dnares/dsaf016)
Supplement: dsaf016_suppl_Supplementary_Table_S1 [file dsaf016_suppl_supplementary_table_s1.docx]

Supplementary Table1 Statistics of reads used

|  | PacBio HiFi reads | Illumina paired end reads |
| --- | --- | --- |
| Number of reads | 3,914,896 | 2,044,104,344 |
| Total size (bp) | 52,127,579,723 | 308,659,755,944 |
